# A Discrete Wavelet Based Feature Extraction and Hybrid Classification Technique for Microarray Data Analysis

**DOI:** 10.1155/2014/195470

**Published:** 2014-08-06

**Authors:** Jaison Bennet, Chilambuchelvan Arul Ganaprakasam, Kannan Arputharaj

**Affiliations:** ^1^Department of Computer Science and Engineering, RMK Engineering College, Anna University, Chennai, India; ^2^Department of Information Science and Technology, Anna University, Chennai, India

## Abstract

Cancer classification by doctors and radiologists was based on morphological and clinical features and had limited diagnostic ability in olden days. The recent arrival of DNA microarray technology has led to the concurrent monitoring of thousands of gene expressions in a single chip which stimulates the progress in cancer classification. In this paper, we have proposed a hybrid approach for microarray data classification based on nearest neighbor (KNN), naive Bayes, and support vector machine (SVM). Feature selection prior to classification plays a vital role and a feature selection technique which combines discrete wavelet transform (DWT) and moving window technique (MWT) is used. The performance of the proposed method is compared with the conventional classifiers like support vector machine, nearest neighbor, and naive Bayes. Experiments have been conducted on both real and benchmark datasets and the results indicate that the ensemble approach produces higher classification accuracy than conventional classifiers. This paper serves as an automated system for the classification of cancer and can be applied by doctors in real cases which serve as a boon to the medical community. This work further reduces the misclassification of cancers which is highly not allowed in cancer detection.

## 1. Introduction

Despite the skill of the doctors or radiologists, there is always a possibility of missing the detection of cancers by using image modalities for the detection of cancers such as breast, lungs, and colon. This is due to various reasons including technical issues in capturing the images, unobservable abnormalities, and misinterpretation of abnormalities. We hereby propose an automated classification system to reduce the diagnosis error using microarray data. The proposed system has the capability to distinguish between normal and abnormal cases of different cancers based on discrete wavelet transform (DWT).

Microarray is the technology for measuring the expression levels of tens of thousands of genes in parallel in a single chip [1–3]. Each chip is about 2 cm by 2 cm and microarrays contain up to 6000 spots. Different microarray technologies include serial analysis of gene expression (SAGE), nylon membrane, and Illumina bead array [4]. Thus microarrays offer an efficient method of gathering data that can be used to determine the expression pattern of thousands of genes. Gene expression data is represented as a matrix in which rows represent genes and columns represent samples or observations. High dimensionality of gene expression data is a big challenge in most of the classification problems. Large number of features (genes) against small sample size and redundancy in expressed data are the two main causes which lead to poor classification accuracy.

Several classification techniques have been employed in the past to deal with microarray data. Reference [5] used a weighted voting scheme, [6] applied support vector machines, and [7] explored several support vector machine techniques, nearest neighbor classifier, and probabilistic neural networks. It has been found that no classification algorithm performs well on all datasets and hence the exploration of several classifiers is useful [8].

Feature selection prior to classification is an essential task. Feature selection methods [9] remove irrelevant and redundant features to improve classification accuracy. Many transformation methods such as independent component analysis [10] and wavelet analysis [11] have also been applied to reduce the dimension of the data in the past. In [12], a feature extraction method based on discrete wavelet transform (DWT) is proposed. The approximation coefficients of DWT together with some useful features from the high frequency coefficients selected by the maximum modulus method are used as features. A novel way to think of microarray data is as a signals set. The number of genes is the length of signals and hence signal processing techniques such as wavelet transform can be used to perform microarray data analysis.

This paper deals with the classification of particular microarray data into normal or abnormal based on discrete wavelet transform (DWT). DWT is an important multiresolution analysis tool that has been commonly applied to signal processing, image analysis, and various classification systems [13]. A novel moving window technique (MWT) is applied for feature extraction and hybrid classifier based on nearest neighbor (NN), naïve Bayes, and support vector machine (SVM) classifier is used for classification purpose. The proposed methods are implemented in MATLAB and the performances of these methods are also analyzed.

The rest of the paper is organized as follows. Microarray and wavelet transforms theoretical framework is discussed in Section 2.  Section 3 describes the development methodology for the classification of microarray data. The classification is achieved by extracting wavelet features based on the proposed MWT. The classifiers used in the proposed methods are NN, Bayes, and SVM. Datasets are described in Section 4.  Section 5 analyzes the experimental results and discussions of the proposed method and finally we draw our conclusions in Section 6.

## 2. Background

### 2.1. Microarray

Large amount of data useful for solving many biological problems can be generated by a technique called microarray. Microarray is a technique which measures the level of activity of thousands of genes concurrently. If the gene is overexpressed then there will be too much protein which gives the conclusion that the particular gene is abnormal. Even much smaller changes can be detected by microarrays compared to karyotypes. The domain where microarray is used in the recent years is in disease classification. Gene expression data is data rich and information poor. Public microarray databases include NCBI, Genbank, Array Express, Gene Expression Omnibus, and Stanford Microarray [14]. Microarray platforms include Agilent, Affymetrix, and Illumina bead array.  Table 1 depicts the gene expression matrix where each cell in the matrix represents the normalized gene expression level.

#### 2.1.1. Steps in Microarray

Listed below is the protocol for microarray technology.

 (1) Collect samples from healthy and cancer patients and keep the samples in two separate tubes.
 (2) Isolate RNA followed by mRNA isolation from healthy and cancerous samples.
 (3) Convert to complimentary DNA (cDNA) with healthy cDNA green in color and cancerous cDNA green in color.
 (4) Apply cDNA from both samples to the microarray.
 (5) Scanning the microarray will tell the difference between infected cell and uninfected cell.

The red spots represent genes that are turned up and the green spots represent genes that are turned down. More mRNA is produced in red spots and less mRNA is produced in green spots. Red spots represent cancer cells and green spots represent normal cells and the yellow spots represent genes that are expressed in both cancer and healthy patients.

### 2.2. Discrete Wavelet Transform

Discrete wavelet transform is a signal processing method by which gene expression data is processed. Wavelet transform is used in gene expression analysis because of its multiresolution approach in signal processing. In this microarray data is transformed into time-scale domain and used as classification features. Gene expression data is represented by a matrix in which rows represent genes and columns represent samples. Since each sample contains thousands of genes, the number of genes can be viewed as the length of the signals. Hence signal processing techniques can be used for microarray data analysis. Wavelets are a family of basis functions. symlet, coiflet, Daubechies, and biorthogonal and reverse biorthogonal wavelets are the wavelet families which are already in use [11]. They vary in various basic properties of wavelets like compactness, smoothness, fast implementation, and orthonormality. One of the key advantages of wavelets is their ability to spatially adapt to features of a function such as discontinuities and varying frequency behavior. The compact support means the localization of wavelets. That is, a region of the data can be processed without affecting the data outside this region.

## 3. The Proposed Method

**Figure 1:**
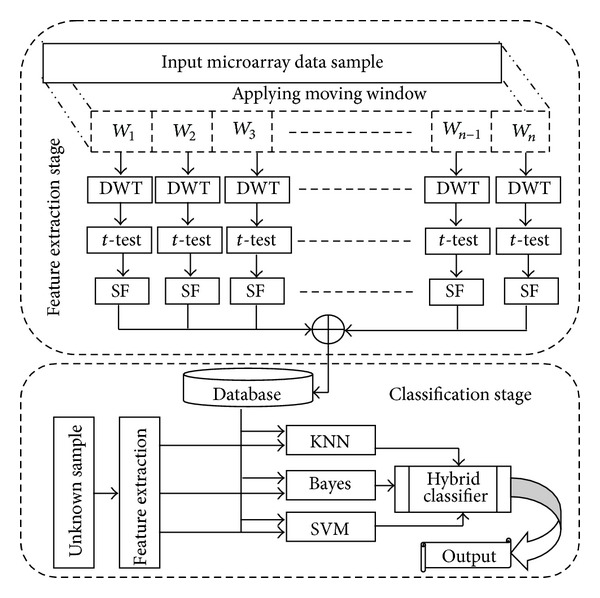
Block diagram of the proposed system.

The main objective of the proposed classification system is to distinguish between the normal and abnormal microarray data of different types of cancer. Five real world microarray datasets used by many researchers are taken to evaluate this study. The two different stages involved in the proposed classification system are feature extraction and classification. They are discussed in the following sections.  Figure 1 shows the block diagram of the proposed system.

### 3.1. Feature Selection

A novel feature extraction technique based on DWT and MWT is proposed. Feature extraction involves simplifying the amount of resources required to describe a large set of data accurately. DWT can be used for high dimensionality data analyses, such as image processing and image data analysis. The proposed method rearranges the data giving a threshold to the wavelet coefficient using DWT and then calculates the approximate value of the raw data after applying an inverse function to the transformed data. After* t*-test, feature selection method is applied to the selected feature at the same approximation value. After ranking the wavelet coefficients inside the window, the top ranked wavelet coefficient is selected as a dominant feature of that window. A similar process is applied for all the windows placed in given microarray data and the resultant top ranked coefficients are stored in the database for further classification. In this paper, the algorithm based on DWT used to perform effective feature selection [9] is shown in Algorithm 1.

The algorithm works as follows. First the window size is defined. Then for each window apply the wavelet transform and define the level of decomposition. It divides into two subbands, namely, scaling coefficient and detailed coefficient, which are called wavelet coefficients. Rearrange the data giving a threshold to the wavelet coefficient and then calculate the approximate value of the raw data after applying an inverse function to the transformed data. *t*-test is applied to select the top ranking features (genes). The algorithm is represented in Algorithm 1.

### 3.2. Classification

Classification of microarray data into normal and abnormal is done by designing hybrid classifier based on NN, naive Bayes, and SVM. In the classification stage, the same kinds of features are extracted and compared with the references obtained in the training stage. Cross-validation is used to estimate how a machine learning algorithm will perform when faced with unfamiliar data. It is intended to reduce error associated with one of the pit falls of machine learning where a hypothesis is formed on the same data to test it. In* K*-fold cross-validation the data is randomly divided into* K* partitions. Data in one partition is used to test and the remaining partitions are used to train. This means that the training data needs to be calculated* K* times as each partition gets tested. In order to evaluate the robustness of the proposed system threefold cross-validation is used. Every fold is used to test the accuracy by using one classifier each. The classification accuracy obtained from each of the classifiers is considered as the weight of the same while the classifiers are hybridized.

## 4. Datasets

To demonstrate the efficiency of our method, the proposed method is evaluated on five gene expression datasets used in the literature.

 (1) Colon dataset: colon dataset [15] is derived from colon cancer patient samples. It consists of the expression levels of 1909 genes of 62 patients among which 40 are colon cancer cases and 22 are normal cases.
 (2) Ovarian dataset: ovarian dataset [16] often serves as benchmark for microarray data analysis in most literatures. The dataset provided here includes 91 controls (normal) and 162 ovarian cancers. There are a total of 15,154 genes.
 (3) CNS dataset: CNS dataset [15] contains 60 patient samples out of which 39 are normal cases and 21 are cancer cases. There are 7129 genes in the dataset.
 (4) Leukemia dataset: leukemia dataset [6] is another dataset widely used in the literature, which is taken as our benchmark dataset. The leukemia dataset contains the expression levels of 7129 genes taken from 72 samples. Labels indicate that there are 47 cancer cases and 25 normal cases.
 (5) Breast dataset: breast dataset [17] consists of 97 samples, of which 51 are normal cases and 46 are cancer cases. Large number of features against small sample size is the trademark of the breast cancer dataset.  Table 2 shows the summary of these datasets.

## 5. Results and Discussion 

In this section, the experimental results and their implications are discussed. Here the performance of the proposed classification system of cancerous microarray data based on DWT and hybrid classifier is explained. To evaluate the performance of the proposed system, computer simulations and experiments with microarray data are performed. The system is implemented in MATLAB version 7.6. The training and testing are run on a modern standard PC (1.66 GHz Intel processor, 1 GB of RAM) running under Windows XP. The metric used to analyze the performance of the proposed system is classification accuracy. In this study, 4 datasets having large number of genes and 1 dataset with minimum number of genes are considered.

As the microarray dataset has different number of samples, 60% of cases are selected for training the classifier and the remaining 40% are used for classification using hybrid classifier. The robustness of the proposed system is evaluated by changing three parameters, namely, different types of wavelet, decomposition level of DWT, and also the window size of the proposed MWT. The types of wavelets used are Daubechies 7 (db7), coiflet 2 (coif2), biorthogonal 2.2 (bior2.2), Symlet 2 (sym2), and reverse biorthogonal 2.2 (rbio2.2), respectively (Coifman and Wickerhauser, 1992). The Daubechies family bases are chosen because of their properties of compact support and orthonormality. The biorthogonal wavelets are chosen for their property of exact reconstruction. The window sizes used in the proposed MWT are 32, 64, 128, 256, 512, and 1024. The performance analysis starts from one-level decomposition to maximum level of decomposition of window size using the wavelet.

Tables 3, 4, 5, 6, and 7 show the window sizes and the various levels in which maximum accuracy is achieved for the proposed system using different types of wavelets for the five benchmark datasets used in our study.

Table 3 reports the classification performance of the individual classifiers with the hybrid classifier for colon dataset using db7. The statistically significant results are in bold face. It is observed that KNN, Bayes, and SVM produce accuracy less than 76 whereas the accuracy of the proposed hybrid classifier for the same dataset is 100% with minimal number of features (four) and for window size 512.

Individual classifiers show considerably very poor performance compared to our hybrid classifier for the breast dataset. The maximum accuracy in terms of individual classifier is 64.44 for KNN in level 1 with a window size of 128. Our ensemble approach produces 100% accuracy with just 24 features for level 4 using window size of 1024. It is obvious from Table 5 to Table 7 that hybrid classifier gives the maximum accuracy compared to the individual classifier.

**Figure 2:**
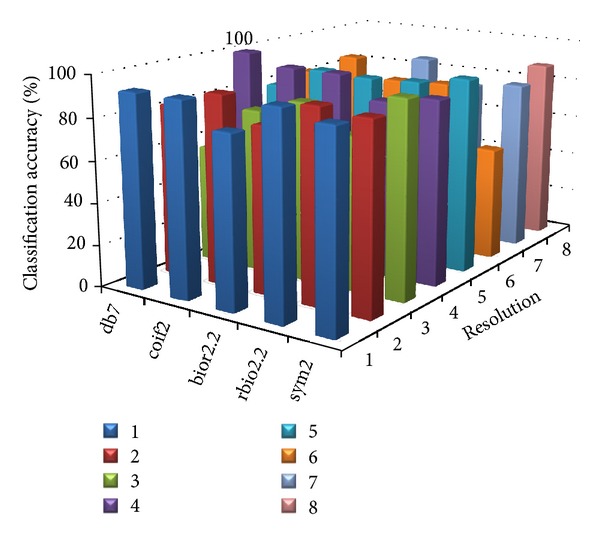
Classification accuracy of the proposed system for breast microarray dataset using window size of 1024.

**Figure 3:**
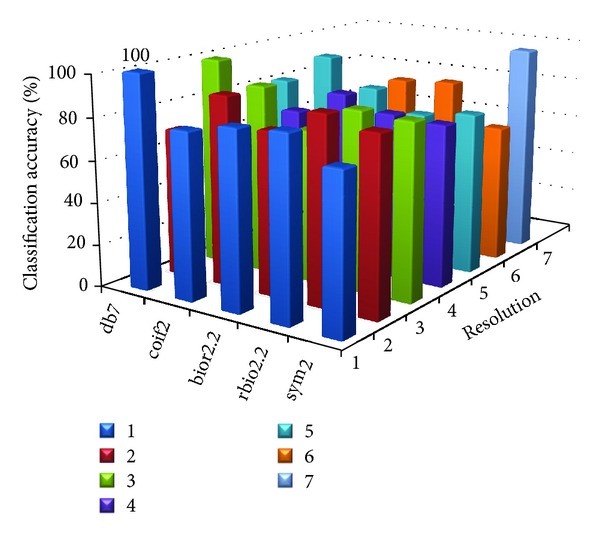
Classification accuracy of the proposed system for colon microarray dataset using window size of 512.

**Figure 4:**
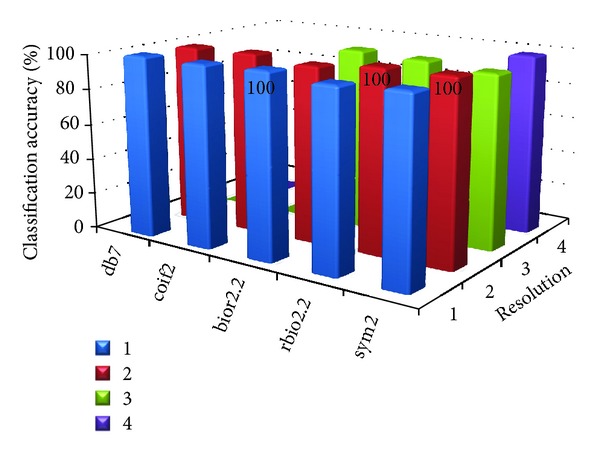
Classification accuracy of the proposed system for ovarian microarray dataset using window size of 64.

**Figure 5:**
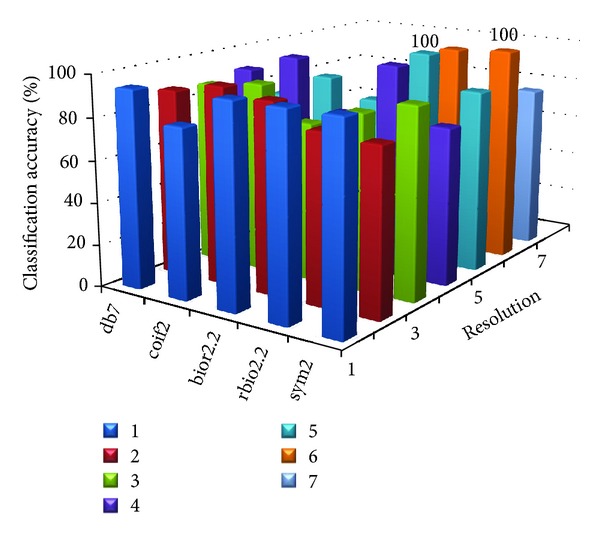
Classification accuracy of the proposed system for CNS microarray dataset using window size of 512.

**Figure 6:**
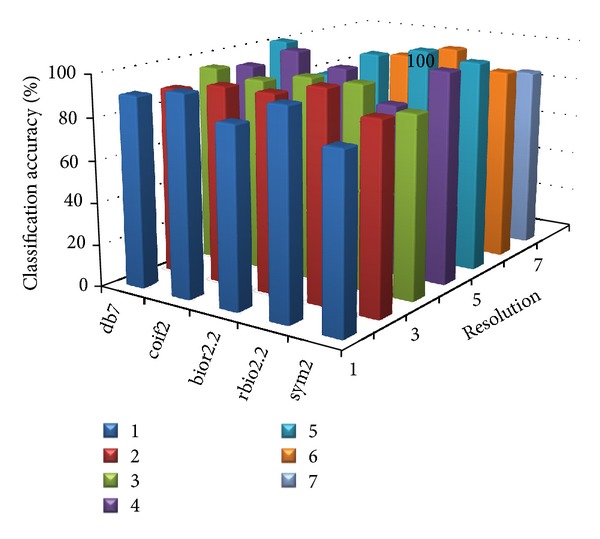
Classification accuracy of the proposed system for Leukemia dataset using window size of 512.

Table 8 shows the parameter value at which the proposed MWT produces the maximum classification accuracy without any misclassification. Figures 2, 3, 4, 5, and 6 show the classification accuracy of the proposed system for breast, colon, ovarian, CNS, and leukemia microarray dataset using the best window size that achieves 100% classification accuracy.

From the figures and Table 8, it is observed that the proposed MWT produces better results with no misclassification and minimum numbers of features are used for the classification.

## 6. Conclusion

In this paper we have proposed a new technique for the classification of microarray data based on DWT. The multiresolutional representation of microarray data is achieved by DWT inside a window of predefined size as test pattern. The performance of the proposed system is assessed by means of extensive computational tests concerning the classification of five cancer microarray datasets: breast, colon, ovarian, CNS, and leukemia. Experimental results show that the proposed method is successful in classifying the microarray data and the successful classification rate is 100% for all five microarray datasets. It is observed from the results that DWT emerges as a potentially dominant feature extraction technique for microarray data classification.

## Figures and Tables

**Algorithm 1:**
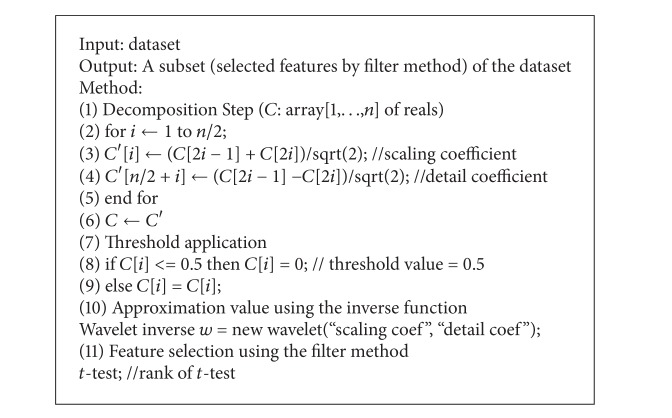
Feature selection method by DWT using scaling coefficient and detailed coefficient.

**Table 1:** *m* samples.

Gene ID	Sample 1	Sample 2	*⋯*	Sample *m*
Gene 1	−1.2	−2.1	3.0	2.9
Gene 2	2.7	0.2	−1.1	1.6
*⋮*	*⋮*	*⋮*	*⋮*	*⋮*
Gene *n*	−2.9	−1.9	2.6	−2.1

**Table 2:** Summary of datasets used for our experimental study.

Dataset	Number of features	Number of samples	Number of normal cases	Number of cancer cases
Breast	24481	97	51	46
Colon	1909	62	22	40
Ovarian	15154	253	91	162
CNS	7129	60	39	21
Leukemia	7129	72	25	47

**Table 3:** Performance analysis of colon dataset using db7 with variations in window sizes and levels.

Classifier	Window size	Classification accuracy (%)					
		Level 1	Level 2	Level 3	Level 4	Level 5	Level 6
KNN	64	**75.65**	75.42	0.00	0.00	0.00	0.00
	128	71.19	60.06	72.75	0.00	0.00	0.00
	256	65.74	53.51	62.72	53.62	0.00	0.00
	512	75.07	41.28	70.96	45.62	68.41	0.00
	1024	64.41	54.84	58.84	64.52	50.72	47.94

Bayes	64	68.52	59.07	0.00	0.00	0.00	0.00
	128	52.06	49.39	60.29	0.00	0.00	0.00
	256	54.96	49.16	56.17	50.72	0.00	0.00
	512	60.41	61.51	42.61	46.49	**71.65**	0.00
	1024	67.07	50.96	64.17	43.71	54.72	59.19

SVM	64	**74.32**	69.97	0.00	0.00	0.00	0.00
	128	60.52	55.07	73.97	0.00	0.00	0.00
	256	65.97	52.06	67.30	63.30	0.00	0.00
	512	64.41	64.17	58.61	38.26	56.29	0.00
	1024	71.30	41.04	71.07	35.48	53.51	56.64

Hybrid	64	96.77	95.16	0.00	0.00	0.00	0.00
	128	98.39	96.77	96.77	0.00	0.00	0.00
	256	85.48	85.48	83.87	82.26	0.00	0.00
	512	**100.00**	69.35	98.39	59.68	80.65	0.00
	1024	75.81	75.81	70.97	95.16	54.84	69.35

**Table 4:** Performance analysis of breast dataset using db7 with variations in window sizes and levels.

Classifier	Window size	Classification accuracy (%)					
		Level 1	Level 2	Level 3	Level 4	Level 5	Level 6
KNN	64	52.22	52.22	0.00	0.00	0.00	0.00
	128	**64.44**	55.56	61.11	0.00	0.00	0.00
	256	55.56	61.11	47.78	57.78	0.00	0.00
	512	50.00	52.22	47.78	48.89	57.78	0.00
	1024	50.00	58.89	41.11	54.44	52.22	58.89

Bayes	64	46.67	52.22	0.00	0.00	0.00	0.00
	128	47.78	47.78	50.00	0.00	0.00	0.00
	256	54.44	**55.56**	53.33	48.89	0.00	0.00
	512	51.11	53.33	53.33	51.11	54.44	0.00
	1024	50.00	54.44	51.11	51.11	54.44	53.33

SVM	64	56.67	52.22	0.00	0.00	0.00	0.00
	128	60.00	50.00	**63.33**	0.00	0.00	0.00
	256	51.11	58.89	61.11	57.78	0.00	0.00
	512	66.67	58.89	60.00	55.56	51.11	0.00
	1024	54.44	60.00	48.89	42.22	53.33	52.22

Hybrid	64	88.66	89.69	0.00	0.00	0.00	0.00
	128	100.00	90.72	100.00	0.00	0.00	0.00
	256	82.47	93.81	78.35	93.81	0.00	0.00
	512	76.29	87.63	83.51	85.57	96.91	0.00
	1024	92.78	82.47	56.70	**100.00**	79.38	82.47

**Table 5:** Performance analysis of CNS dataset using rbio2.2 with variations in window sizes and levels.

Classifier	Window size	Classification accuracy (%)						
		Level 1	Level 2	Level 3	Level 4	Level 5	Level 6	Level 7
KNN	64	57.46	55.41	60.67	0.00	0.00	0.00	0.00
	128	58.11	54.68	53.29	58.55	0.00	0.00	0.00
	256	55.41	63.74	47.73	59.94	58.92	0.00	0.00
	512	64.11	54.39	48.76	60.96	**70.03**	62.72	0.00
	1024	59.28	55.77	59.58	48.76	62.72	54.68	50.88

Bayes	64	50.15	63.01	67.91	0.00	0.00	0.00	0.00
	128	60.23	57.82	69.30	67.62	0.00	0.00	0.00
	256	54.31	51.54	49.12	**70.76**	56.43	0.00	0.00
	512	59.21	59.94	53.29	53.29	59.21	59.94	0.00
	1024	54.02	48.46	62.72	50.88	58.55	54.68	55.77

SVM	64	62.72	63.74	65.20	0.00	0.00	0.00	0.00
	128	**73.54**	64.11	57.82	59.94	0.00	0.00	0.00
	256	59.58	52.63	47.00	64.47	51.61	0.00	0.00
	512	51.24	48.76	57.82	45.98	60.60	49.49	0.00
	1024	49.49	57.09	67.25	48.39	69.37	47.73	39.69

Hybrid	64	100.00	90.00	95.00	0.00	0.00	0.00	0.00
	128	100.00	96.67	86.67	75.00	0.00	0.00	0.00
	256	96.67	96.67	95.00	90.00	90.00	0.00	0.00
	512	93.33	78.33	81.67	98.33	**100.00**	98.33	0.00
	1024	90.00	70.00	86.67	73.33	73.33	86.67	70.00

**Table 6:** Performance analysis of leukemia set using rbio2.2 with variations in window sizes and levels.

Classifier	Window size	Classification accuracy (%)						
		Level 1	Level 2	Level 3	Level 4	Level 5	Level 6	Level 7
KNN	64	81.56	83.86	88.21	0.00	0.00	0.00	0.00
	128	81.26	82.41	87.61	83.56	0.00	0.00	0.00
	256	68.57	62.52	74.06	81.56	**94.25**	0.00	0.00
	512	63.97	74.91	62.22	61.97	88.21	89.36	0.00
	1024	60.22	75.51	60.17	83.01	59.37	85.31	56.47

Bayes	64	**92.80**	91.35	86.46	0.00	0.00	0.00	0.00
	128	81.01	85.31	90.75	86.46	0.00	0.00	0.00
	256	70.91	92.50	77.81	80.11	87.61	0.00	0.00
	512	83.01	72.86	69.17	68.62	83.86	91.35	0.00
	1024	64.27	81.86	65.97	87.91	61.12	85.61	61.37

SVM	64	91.35	87.31	**92.80**	0.00	0.00	0.00	0.00
	128	87.06	78.41	92.50	85.31	0.00	0.00	0.00
	256	72.91	85.86	73.46	77.51	68.62	0.00	0.00
	512	74.91	75.21	63.72	63.72	83.61	84.16	0.00
	1024	67.72	80.71	61.92	82.71	64.57	85.31	59.37

Hybrid	64	98.61	100.00	100.00	0.00	0.00	0.00	0.00
	128	100.00	90.28	98.61	97.22	0.00	0.00	0.00
	256	95.83	95.83	90.28	100.00	100.00	0.00	0.00
	512	93.06	95.83	93.06	79.17	**100.00**	97.22	0.00
	1024	76.39	88.89	79.17	93.06	83.33	94.44	76.39

**Table 7:** Performance analysis of ovarian dataset using sym2 with variations in window sizes and levels.

Classifier	Window size	Classification accuracy (%)							
		Level 1	Level 2	Level 3	Level 4	Level 5	Level 6	Level 7	Level 8
KNN	64	91.31	**97.28**	91.36	91.04	0.00	0.00	0.00	0.00
	128	94.08	87.99	87.66	70.92	85.49	0.00	0.00	0.00
	256	88.64	73.05	82.50	71.84	67.43	80.61	0.00	0.00
	512	84.62	53.85	74.50	64.07	65.98	62.61	72.88	0.00
	1024	68.31	69.62	65.81	60.75	78.27	65.21	57.23	61.96

Bayes	64	89.35	**96.20**	90.98	91.69	0.00	0.00	0.00	0.00
	128	80.07	82.78	88.32	93.10	89.95	0.00	0.00	0.00
	256	82.45	83.91	83.53	79.08	87.54	84.79	0.00	0.00
	512	83.76	80.47	82.83	74.67	78.96	73.74	75.00	0.00
	1024	71.73	69.36	79.95	63.85	84.41	70.82	69.03	67.77

SVM	64	90.00	93.75	92.72	83.65	0.00	0.00	0.00	0.00
	128	85.01	90.60	86.03	**95.54**	90.33	0.00	0.00	0.00
	256	80.82	83.33	88.97	81.74	86.68	78.31	0.00	0.00
	512	72.52	82.98	76.63	70.82	76.36	77.98	73.04	0.00
	1024	61.20	60.31	80.35	69.12	81.86	67.22	60.49	62.17

Hybrid	64	98.81	**100.00**	95.26	98.81	0.00	0.00	0.00	0.00
	128	99.21	95.65	98.81	96.44	94.47	0.00	0.00	0.00
	256	99.21	94.47	89.33	87.35	91.30	93.68	0.00	0.00
	512	96.84	86.96	92.09	87.75	88.14	81.82	85.77	0.00
	1024	84.98	84.98	85.38	72.73	89.72	79.45	81.03	84.98

**Table 8:** Parameter settings to achieve 100% classification accuracy.

Dataset	Wavelet	Level	Best window size	Number of features used
Breast	db7	4	1024	24
Colon	db7	1	512	4
Ovarian	sym2, bior2.2, rbio2.2	2	64	237
CNS	rbio2.2, sym2	5	512	14
Leukemia	rbio2.2	5	512	14
